# Case Report: Complete pathologic response to neoadjuvant selpercatinib in a patient with resectable early-stage *RET* fusion-positive non-small cell lung cancer

**DOI:** 10.3389/fonc.2023.1178313

**Published:** 2023-05-18

**Authors:** Jonathan W. Goldman, Lynette M. Sholl, Sanja Dacic, Michael C. Fishbein, Yonina R. Murciano-Goroff, Ravi Rajaram, Sylwia Szymczak, Anna M. Szpurka, Bo H. Chao, Alexander Drilon

**Affiliations:** ^1^ David Geffen School of Medicine, University of California, Los Angeles, Los Angeles, CA, United States; ^2^ Department of Pathology, Brigham and Women’s Hospital and Harvard Medical School, Boston, MA, United States; ^3^ Department of Pathology, Yale School of Medicine, New Haven, CT, United States; ^4^ Department of Medicine, Memorial Sloan-Kettering Cancer Center New York, NY, United States; ^5^ Department of Thoracic and Cardiovascular Surgery, The University of Texas MD Anderson Cancer Center, Houston, TX, United States; ^6^ Loxo@Lilly, Eli Lilly and Company, Warsaw, Poland; ^7^ Loxo@Lilly, Eli Lilly and Company, Indianapolis, IN, United States; ^8^ Loxo@Lilly, Eli Lilly and Company, New York, NY, United States; ^9^ Department of Medicine, Weill Cornell Medical College, New York, NY, United States

**Keywords:** RET fusion, selective RET inhibitor, NSCLC, neoadjuvant, major pathologic response, pathologic complete response, case report

## Abstract

The LIBRETTO-001 trial demonstrated the activity of the selective rearrangement during transfection (RET) inhibitor selpercatinib in advanced *RET* fusion-positive non-small cell lung cancer (NSCLC) and resulted in the drug’s approval for this indication. A cohort that included neoadjuvant and adjuvant selpercatinib was opened on LIBRETTO-001 for early-stage *RET* fusion-positive NSCLC with the primary endpoint of major pathologic response. A patient with a stage IB (cT2aN0M0) *KIF5B-RET* fusion-positive NSCLC received 8 weeks of neoadjuvant selpercatinib at 160 mg twice daily followed by surgery. While moderate regression in the primary tumor (stable disease, Response Evaluation Criteria in Solid Tumors (RECIST) guidelines version 1.1) was observed radiologically, assessment via an Independent Pathologic Review Committee revealed a pathologic complete response (0% viable tumor). This consensus assessment by three independent pathologists was aided by *RET* fluorescence *in situ* hybridization testing of a reactive pneumocyte proliferation showing no rearrangement. Neoadjuvant selpercatinib was well-tolerated with only low-grade treatment-emergent adverse events. The activity of prospective preoperative selpercatinib in this case establishes proof of concept of the potential utility of RET inhibitor therapy in early-stage *RET* fusion-positive NSCLC.

## 1 Introduction

Although cytotoxic chemotherapy and immunotherapy remain cornerstones of perioperative therapy in non-small cell lung cancer (NSCLC), many patients experience recurrent disease and limited survival. In trials randomizing epidermal growth factor receptor (*EGFR*)-mutant NSCLC to targeted therapy versus chemotherapy, neoadjuvant EGFR tyrosine kinase inhibitor (TKI) achieved high objective response rates (42%–62%), major pathologic response (MPR; 8%–24%), and prolonged progression-free survival (1). Neoadjuvant anaplastic lymphoma kinase TKI therapy was likewise found to be active in *anaplastic lymphoma kinase* fusion-positive NSCLC, including in patients for whom pathologic complete response (pCR) was achieved (2).

**Figure 1:**
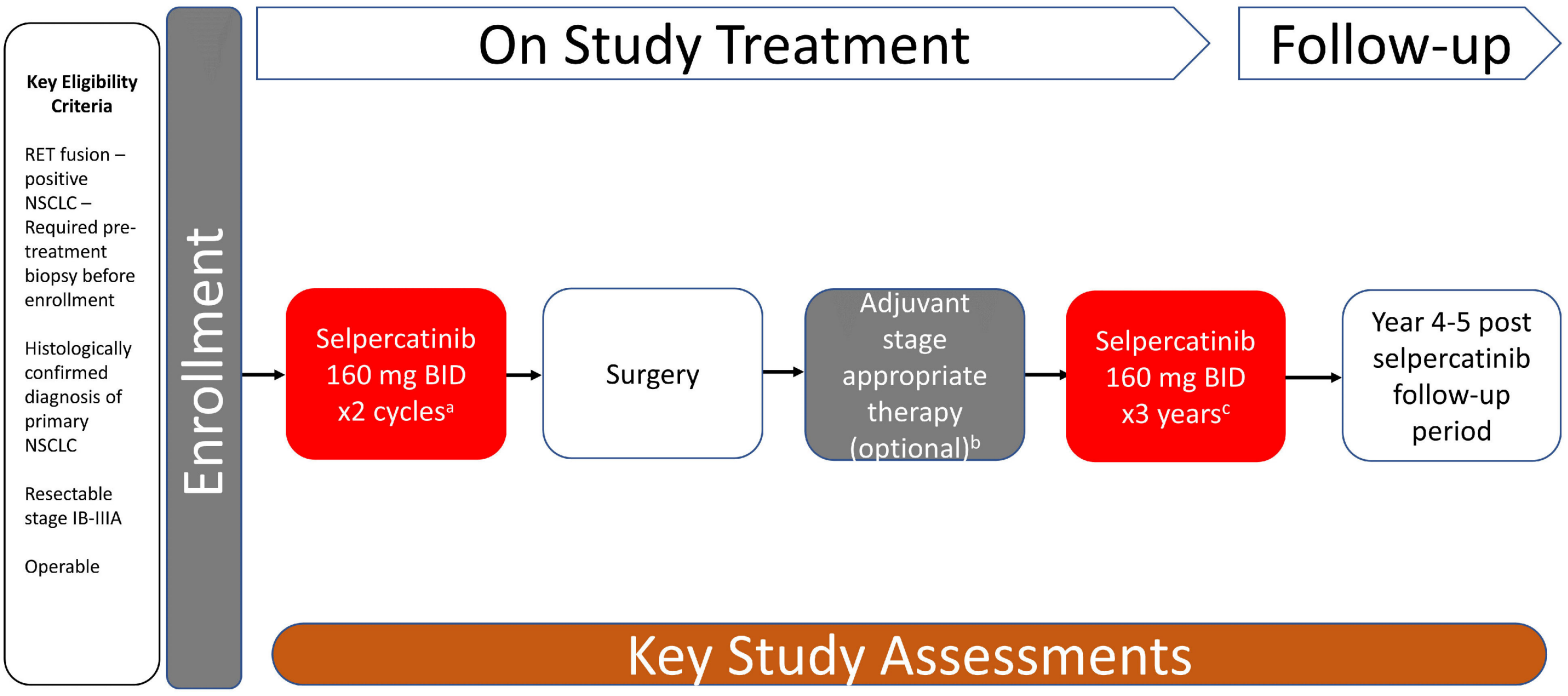
Study design. LIBRETTO-001 trial is a phase 2, open-label, single-arm study of selpercatinib for patients with RET-dependent cancer. The study design of the neoadjuvant-adjuvant cohort (cohort 7) of the trial is summarized in this figure. ^a^ Each cycle is 28 days. ^b^ Treating physician’s decision. ^c^ Up to 3 years of treatment or disease recurrence, patient withdrawal, intolerable toxicity, or death, whichever occurs first. BID, twice daily; NSCLC, non-small cell lung cancer; RET, rearrangement during transfection.

We thus prospectively explored the neoadjuvant use of selpercatinib, a highly selective and potent rearrangement during transfection (RET) kinase inhibitor with central nervous system activity approved in multiple countries for the treatment of *RET*-altered advanced or metastatic lung or thyroid cancers. The LIBRETTO-001 study (NCT03157128; which provided registration data resulting in the aforementioned approvals) was formally amended in 2021 to include neoadjuvant and adjuvant selpercatinib for early-stage *RET* fusion-positive NSCLC (3) with the primary endpoint of MPR (**Figure 1**).

## 2 Case report

**Figure 2:**
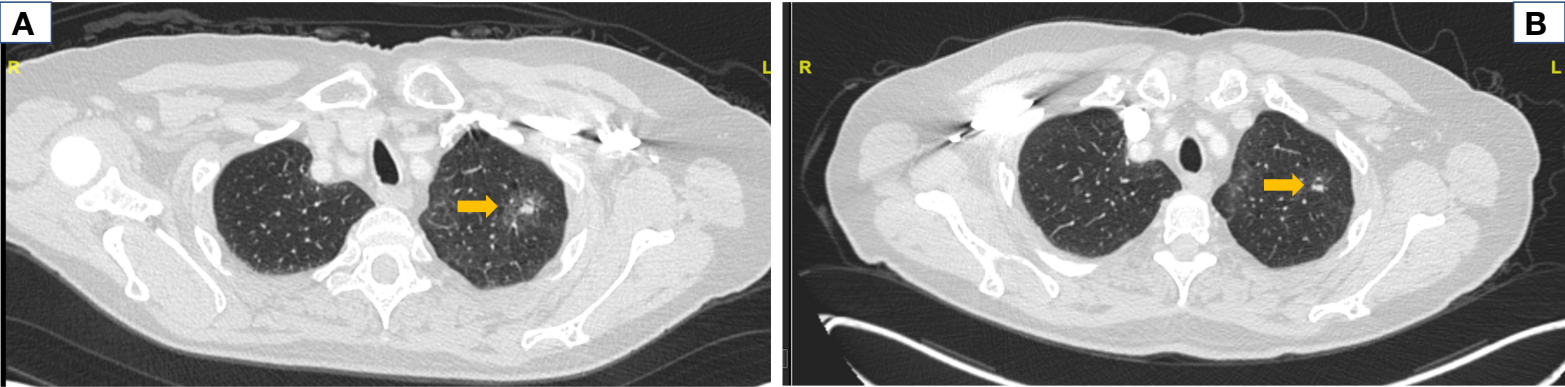
Radiologic response to neoadjuvant selpercatinib. Computed tomography (CT) imaging of chest before and after neoadjuvant selpercatinib treatment is shown. **(A)** A baseline CT of the chest before selpercatinib initiation revealed a left lung mass measuring 3.1 cm (arrow). **(B)** A follow-up CT of the chest after 8 weeks of selpercatinib showed disease regression to 2.9 cm in the same mass (arrow). This was classified as stable disease by RECIST v1.1 per investigator assessment. RECIST, Response Evaluation Criteria in Solid Tumors.

**Figure 3:**
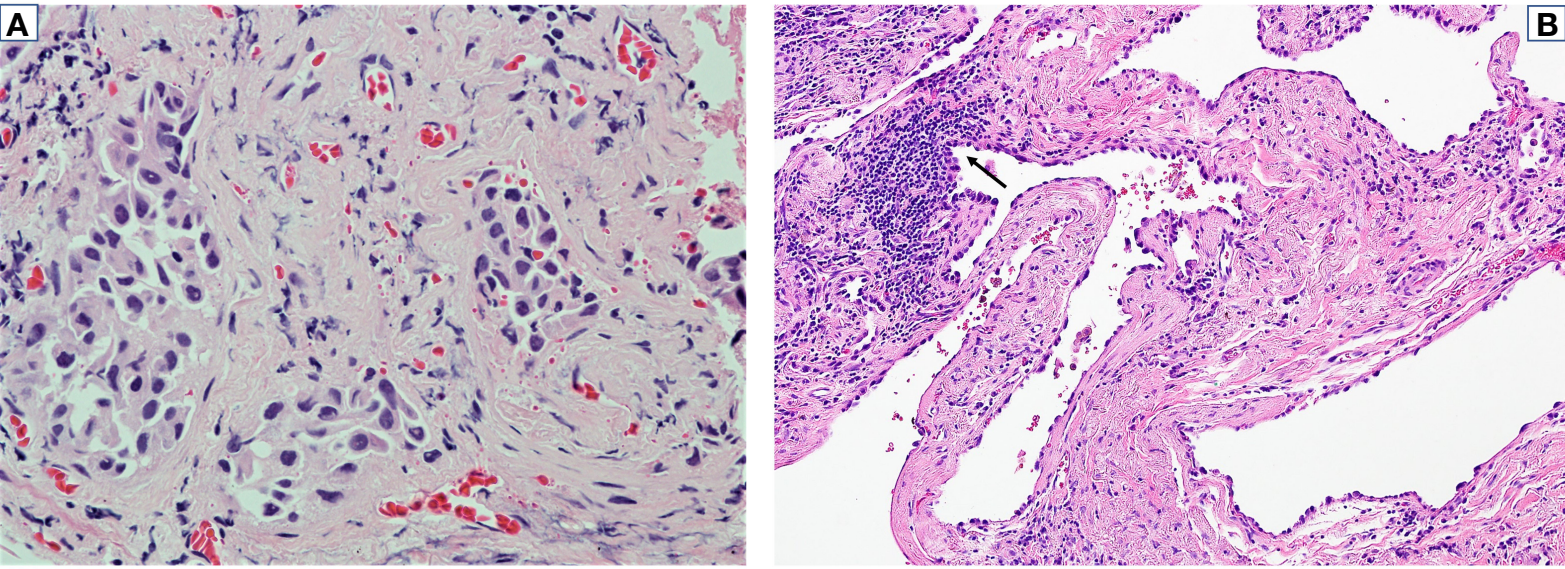
Pathologic response to neoadjuvant selpercatinib. Tumor specimens before and after neoadjuvant selpercatinib treatment are shown. **(A)** Pathologic assessment of a bronchoscopic biopsy with H&E staining before selpercatinib treatment identified a lung adenocarcinoma (×400). **(B)** Resected tumor after 8 weeks of selpercatinib treatment showed no evidence of viable tumor cells (×200). Reactive pneumocyte proliferation is denoted by an arrow. Cytogenetic testing of this specimen via *RET* FISH was negative for a *RET* fusion. FISH, fluorescence *in situ* hybridization.

A 76-year-old Caucasian woman with a remote, five pack-year history of former smoking was diagnosed with clinical stage IB (T2aN0M0) adenocarcinoma of the lung at the University of California, Los Angeles, in September 2021. Baseline radiologic tumor assessments identified a left upper lobe tumor (3.1 cm, **Figure 2**) and no evidence of lymph node or distant metastasis; no F-fluorodeoxyglucose avidity was observed. A transbronchial tumor biopsy showed adenocarcinoma of the lung (**Figure 3**). DNA- and RNA-based next-generation sequencing of 648 tumor-associated genes (Tempus xT™ Version 4) identified a *KIF5B-RET* fusion and no other activating genomic aberrations.

Resectability was confirmed by a thoracic surgeon, and the patient consented to LIBRETTO-001 in October 2021, including the consent to publish her study-related information while keeping her identity confidential. In November 2021, neoadjuvant selpercatinib was initiated at the recommended dose of 160 mg twice daily for two 28-day cycles. Treatment was well tolerated; the only treatment-emergent adverse events (TEAEs) were grade 1 fatigue and diarrhea and grade 2 hypertension. During this time, one dose was missed inadvertently, and there was no dose modification planned.

After the completion of neoadjuvant selpercatinib, the tumor decreased from 3.1 to 2.9 cm (Response Evaluation Criteria in Solid Tumors (RECIST) 1.1 stable disease, investigator assessment, **Figure 2**). In early January 2022, the patient underwent left upper lobe apical and posterior segmentectomies. The surgery was performed as planned; no surgery-related adverse events were documented. One lymph node in levels 5–6, one lymph node in level 7, and fragments of lymph node(s) from left levels 11, 12, and 13 stations were all negative for carcinoma. The patient declined adjuvant selpercatinib due to low-grade TEAEs during neoadjuvant therapy.

The primary endpoint of the LIBRETTO-001 cohort was MPR. Pathologic assessment of the resected primary tumor in hematoxylin and eosin (H&E) staining was conducted by three independent pathologists including two from the Independent Pathologic Review Committee. On initial assessment, 0%, 10%, and 30% viable tumor cells were found.

To adjudicate the presence or absence of viable tumor cells, dual-color break-apart RET (10q11) fluorescence *in situ* hybridization (FISH) was performed (University of California, Los Angeles, Clinical Laboratory), which did not detect aberrations in the 200 nuclei examined. Alignment on the pathologic assessment of 0% viable tumor cells (pCR) was then reached among the three pathologists. The consensus was atypical cells represented a reactive pneumocyte proliferation (instead of carcinoma) secondary to treatment effect (**Figure 3**).

Plasma circulating tumor DNA samples were collected. *KIF5B-RET* was undetectable (Guardant360) in plasma throughout the trial (pre-selpercatinib, pre-operatively, and post-operatively) likely due to the fact that fusions are less well-detected by circulating tumor DNA and early-phase disease may have limited shedding. No other significant alterations were identified; a *platelet-derived growth factor receptor alpha* V193I variant of uncertain significance was found pre-selpercatinib (0.19% allele frequency) but not thereafter. This mutation was not detected by next-generation sequencing of the pre-selpercatinib lung cancer biopsy.

## 3 Discussion

In this patient with a *KIF5B-RET*-positive stage IB lung adenocarcinoma, prospective treatment with neoadjuvant selpercatinib in the LIBRETTO-001 trial achieved pCR. This case provides the first proof of concept of the clinical activity of a RET TKI in the neoadjuvant setting and sets a precedent for ongoing and future investigations in early-stage disease. While this cohort was closed after this patient was treated (for reasons unrelated to safety or activity), a separate trial continues to explore the utility of selpercatinib as adjuvant therapy in *RET* fusion-positive NSCLC (LIBRETTO-432; NCT04819100) (4).

pCR is an accepted regulatory endpoint that has supported the approval for neoadjuvant systemic therapy in NSCLC. Neoadjuvant nivolumab and chemotherapy were approved based on pCR as a main outcome measure (5). While it remains to be seen if RET inhibition will eventually be approved as perioperative therapy for RET fusion-positive NSCLC, neoadjuvant TKI therapy has already been approved for another fusion-positive subset of non-metastatic cancer (i.e., tropomyosin receptor kinase (TRK) inhibition for NTRK fusion-positive cancer, including NSCLC, one of whose approved indications is the setting where surgical resection likely results in severe morbidity) (6).

Several other factors are notable in this case. The discordance between radiologic (stable disease) and pathologic (pCR) assessments, conducted within a week of each other, highlights the inherent limitations of RECIST and the importance of pathologic evaluation in fully demonstrating the tumor response to effective therapy, especially in the short timelines of neoadjuvant therapy. Pathologic response in the setting of minimal radiologic response may need to be considered in other settings as well where surgical resection is not performed.

This case also highlights interobserver variation in the interpretation of pathologic response due to the challenge of using standard H&E staining to determine reactive versus neoplastic epithelium, especially in the setting of lepidic proliferations. To mitigate this challenge, molecular adjudication via FISH was utilized (7). A negative RET FISH result provided evidence of the lack of residual tumor (at least related to the original clone) and helped determine that the initially perceived “viable tumor cells” by two of three pathologists represented a benign reactive proliferation (8). The absence of driver alterations suggests a lack of residual tumor since driver alterations are typically retained during the course of cancer evolution (9). The scenario where the original driver is “lost” likely indicates that an independent clone (not related to the tumor in question) has become dominant. This scenario is unlikely in the present case but cannot be entirely ruled out; however, limited tissue precludes further analysis to address this question.

In summary, neoadjuvant selpercatinib was found to be active for a patient with *RET* fusion-positive NSCLC. Neoadjuvant therapy resulted in low-grade TEAEs but did not preclude or interfere with the performance of definitive surgical therapy. The activity of preoperative selpercatinib in this prospective case establishes proof of concept of the potential utility of selpercatinib in early-stage *RET* fusion-positive NSCLC.

## Data availability statement

The raw data supporting the conclusions of this article will be made available by the authors, without undue reservation.

## Ethics statement

The studies involving human participants were reviewed and approved by UCLA Institutional Review Board (UCLA IRB) – Office of the Human Research Protection Program (OHRPP). The patients/participants provided their written informed consent to participate in this study. Written informed consent was obtained from the individual(s) for the publication of any potentially identifiable images or data included in this article.

## Author contributions

This study was designed jointly by all authors. The sponsor (Loxo Oncology) collected, analyzed, and interpreted the trial data in collaboration with the authors. All authors were involved in the writing of the manuscript and its revisions. All the authors vouch for the completeness and accuracy of the clinical data and analyses and for the adherence of the trial to the protocol. All authors contributed to the article and approved the submitted version.
